# The rapid growth of a pleomorphic adenoma of the parotid gland in the third trimester of pregnancy

**DOI:** 10.1186/1752-1947-5-141

**Published:** 2011-04-09

**Authors:** Frederike Palluch, Martin Lehmann, Joachim Volz, Tahwinder Upile, Holger Sudhoff

**Affiliations:** 1 Department of Otorhinolaryngology, Head and Neck Surgery Klinikum Bielefeld, Bielefeld, Germany; 2 Department of Gynecology and Obstetrics, Klinikum Bielefeld, Bielefeld, Germany; 3 Visiting Fellow from Department of Otorhinolaryngology, Head and Neck Surgery Chase Farm & Barnet Hospitals, UK

## Abstract

**Introduction:**

We report a case highlighting the multidisciplinary management of a giant pleomorphic adenoma of the parotid gland that showed rapid growth in the third trimester of pregnancy.

**Case presentation:**

A 43-year-old Caucasian woman presented in her 32nd week of gestation with a tumor of the parotid gland. Ultrasonography of her neck showed a parotid lesion of 40 × 30 × 27.5 mm. A follow-up magnetic resonance imaging scan of the neck four weeks later revealed that the tumor had grown to 70 × 60 × 60 mm, reaching the parapharyngeal space with marked obstruction of the oropharynx of about 50%. After discussing the case with our multidisciplinary tumor board and the gynecologists it was decided to deliver the baby by caesarean section in the 38th week of gestation, and then to perform a surgical resection of the tumor.

**Conclusion:**

Indications for early surgical intervention of similar cases should be discussed on an individual patient basis in a multidisciplinary setting.

## Introduction

With an incidence of 65%, pleomorphic adenoma is the most common tumor of the salivary glands [1], and 80% of the pleomorphic adenomas are located in the parotid gland. These tumors are characterized by slow growth over a period of years, and tend to remain asymptomatic. In 4% of cases, they turn into malignant tumors. In the following case report, we describe a pleomorphic adenoma of the parotid gland that showed rapid growth within the third trimester of pregnancy.

### Case presentation

A 43-year-old Caucasian woman presented in her 32nd week of gestation with a tumor of the parotid gland. She had noticed the swelling increasing over the past six months. She was otherwise asymptomatic, without any pain, mouth-opening difficulties or facial nerve dysfunction. Ultrasonography of her neck showed a parotid lesion 40 × 30 × 27.5 mm in size, with an heterogeneous appearance. Considering her advanced state of pregnancy, we advised conservative management in the form of watchful waiting and regular review until the birth.

**Figure 1:**
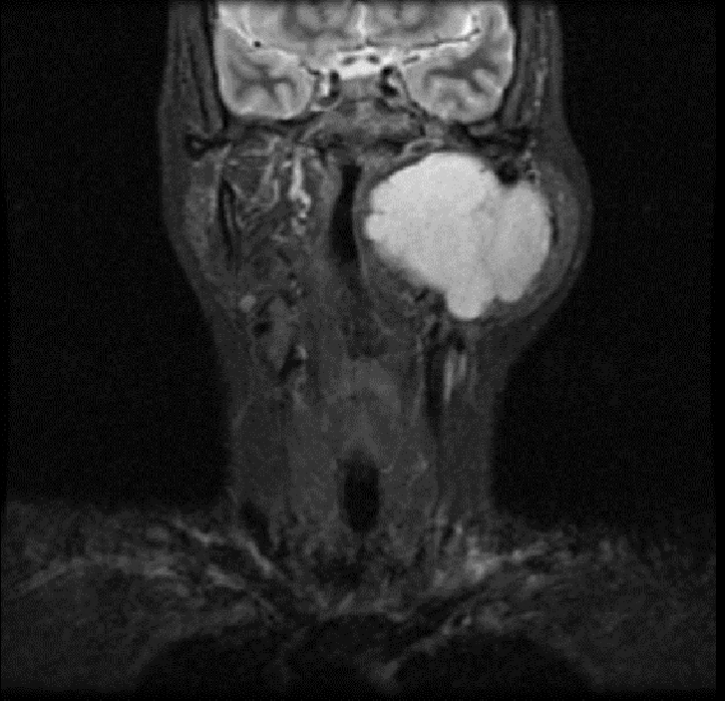
**A coronal T2-weighted MRI of the head and neck region (36th week of pregnancy)**. The left parotid mass is seen distorting the pharynx. The outline of the heterogeneous lesion is clearly demarcated, and tissue planes preserved.

**Figure 2:**
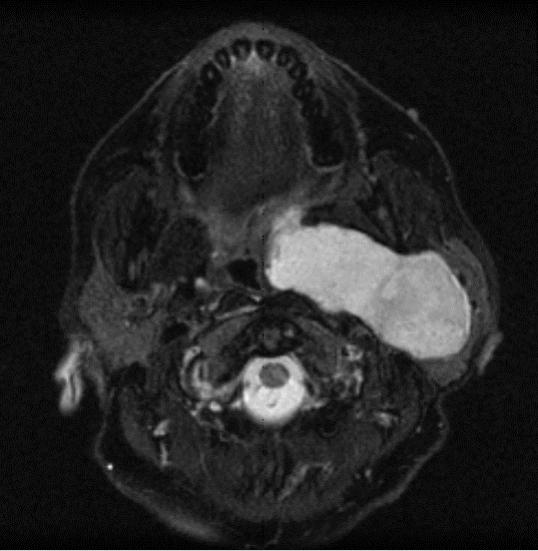
**An axial T2-weighted MRI of the head and neck region (36th week of pregnancy)**. The outline of the heterogeneous left parotid lesion is again clearly demarcated and tissue planes preserved. The oropharyngeal lumen is distorted.

At our patient's next review, only five weeks later, we noticed rapid growth of the lesion with expansion into the parapharyngeal space. There was still no facial nerve involvement, although our patient's mouth opening was slightly limited. Magnetic resonance imaging (MRI) of the neck showed that the tumor had increased to 70 × 60 × 60 mm, reaching the parapharyngeal space with marked obstruction of the oropharynx of about 50% (Figures 1, 2). Further diagnostic tests such as fine-needle aspiration cytology (FNAC) were discussed. We were concerned about the risk of malignancy and increasing local complications due to the rapid growth. Considering the risk of spreading tumor cells and the unreliability of FNAC in identifying the malignant nature of parotid carcinoma [2], we decided to perform surgical resection to prevent further complications and to obtain a secure diagnosis.

After discussing this case with the gynecologists at our interdisciplinary tumor board meeting, it was decided to deliver the baby by caesarean section in the 38th week of gestation with epidural anesthesia using mepivacain and sufentanil. The surgical resection of the tumor was performed four days later. It was possible to completely remove the tumor via a standard cervicofacial incision without the need to resect the mandible. Although the tumor had stretched the facial nerve to double its usual length, there was no postoperative nerve dysfunction (House Brackmann grade I).

**Figure 3:**
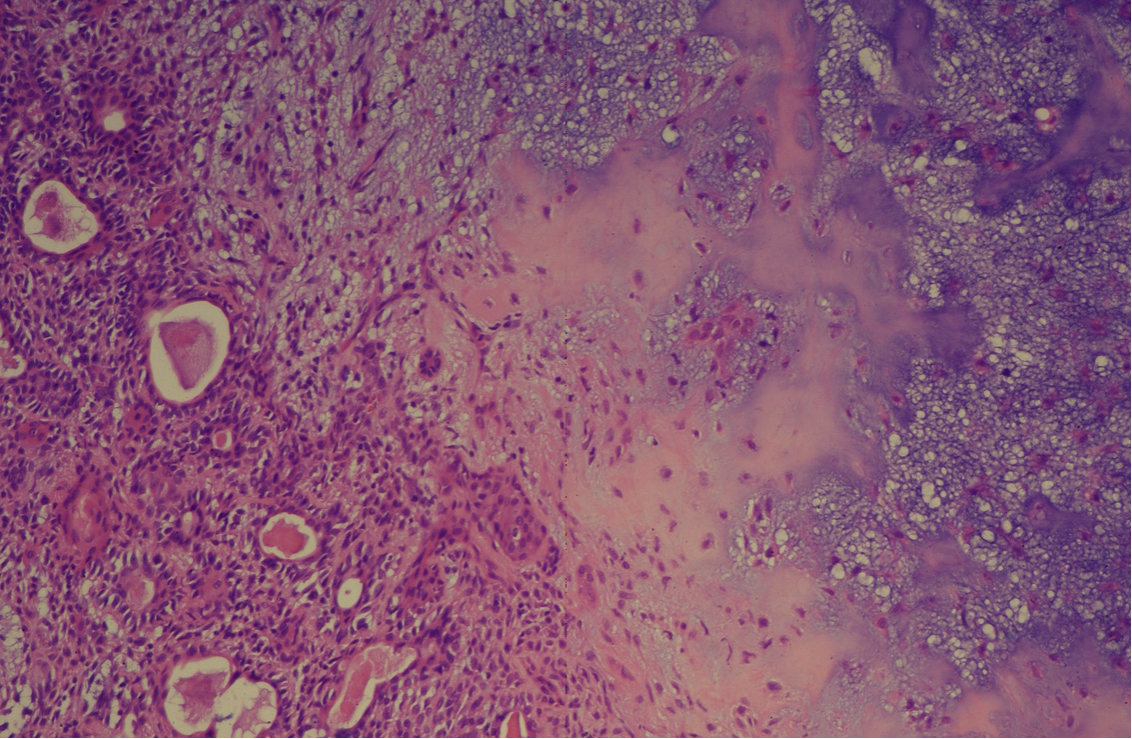
**Pleimorphic adenoma without evidence of malignancy (hematoxylin and eosin, original magnification × 100)**.

On histological analysis, the lesion was identified as a pleomorphic adenoma without evidence of malignancy (Figure 3). Immunohistochemistry did not show any positive expression of oestrogen or progesterone receptors. Our patient remained well with no evidence of recurrence after a follow-up of one year.

## Discussion

The incidence of head and neck cancers during pregnancy is rising [3]. Pleomorphic adenomas are characterized by slow growth over a term of several years. Rapid increase or the appearance of facial nerve dysfunction may indicate malignant transformation, which has been reported in 4% of cases. Rapid growth during the third trimester of pregnancy may indicate a possible hormonal influence on pleomorphic adenomas. A scientifically based relationship is not proven [4-8]. There may be a prognostic association between the expression of progesterone receptors and recurrent pleomorphic adenoma of the parotid gland [9]. Although in this case described here, we could not establish such a relationship, it is possible that unknown factors (e.g. insulin-like growth factor, vascular endothelial growth factor human placental lactogen).are released by the fetoplacental unit, and that these stimulate growth in other end organs such as the parotid

## Conclusion

We recommend frequent monitoring of tumors of the salivary glands diagnosed during pregnancy. Indications for early surgical intervention should be discussed on a individual patient basis in a multidisciplinary setting.

## Consent

Written informed consent was obtained from the patient for publication of the manuscript and accompanying images. A copy of the written consent is available for review by the Editor-in-Chief of this journal.

## Competing interests

The authors declare that they have no competing interests.

## Authors' contributions

FP was the major contributor in writing the manuscript. ML helped to assemble the data and assisted with the manuscript writing. JV provided expert advice, performed the Caesarean section, and revised his part of the manuscript. TU helped to revise the manuscript, HS performed the gland surgery and performed the final reading of the manuscript. All authors have read and approved the final manuscript.
